# COVID-19: Behandlungsstrategien der deutschsprachigen Kinderrheumatologen: Ergebnisse einer Online-Umfrage

**DOI:** 10.1007/s00393-020-00854-8

**Published:** 2020-08-18

**Authors:** A. Janda, C. Schuetz, M. Heeg, K. Minden, C. M. Hedrich, T. Kallinich, C. Hinze, A. Schulz, F. Speth

**Affiliations:** 1 Klinik für Kinder- und Jugendmedizin, Universitätsklinikum Ulm, Eythstr. 24, 89075 Ulm, Deutschland; 2 Klinik und Poliklinik für Kinder- und Jugendmedizin, Universitätsklinikum Carl Gustav Carus an der Technischen Universität Dresden, Dresden, Deutschland; 3 Institut für Immundefizienz und Zentrum für Kinder- und Jugendmedizin, Universitätsklinikum Freiburg, Freiburg, Deutschland; 4 Charité Universitätsmedizin Berlin und Deutsches Rheuma-Forschungszentrum Berlin, Berlin, Deutschland; 5 Department of Women’s & Children’s Health, Institute of Life Course and Medical Sciences, University of Liverpool & Department of Paediatric Rheumatology, Alder Hey Children’s NHS Foundation Trust Hospital, Liverpool, Großbritannien; 6 Klinik für Pädiatrische Rheumatologie und Immunologie, Universitätsklinikum Münster, Münster, Deutschland; 7 Zentrum für Geburtshilfe, Kinder- und Jugendmedizin, Sektion Pädiatrische Stammzelltransplantation und Immunologie, Abteilung Kinderrheumatologie, Universitätsklinikum Hamburg-Eppendorf, Hamburg, Deutschland

**Keywords:** Kinder, Rheumatologie, COVID-19, Therapie, SARS-CoV‑2, Children, Rheumatic diseases, COVID-19, Therapy, SARS-CoV‑2

## Abstract

**Hintergrund:**

Zuverlässige Daten zu Verlauf und Therapie von COVID-19 („corona virus disease 2019“) bei Kindern mit rheumatischen Erkrankungen unter Immunsuppression fehlen.

**Ziel der Arbeit:**

Abbildung individueller Strategien der Mitglieder der Gesellschaft für Kinder- und Jugendrheumatologie (GKJR) im Umgang mit COVID-19.

**Methodik:**

Mittels Online-Umfrage wurden im Mai 2020 das Meinungsbild der GKJR-Mitglieder zum Umgang mit DMARDs („disease-modifying anti-rheumatic drugs“) bei COVID-19-Erkrankung sowie die Bereitschaft zum Einsatz spezieller Therapieansätze bei Patienten mit unterschiedlicher Schwere von COVID-19 erhoben.

**Ergebnisse:**

Es nahmen 71 Kollegen (27,3 % aller befragten ärztlichen Mitglieder) an der Umfrage teil; davon hatten 28,2 % bereits Patienten mit COVID-19 betreut. Über 95 % der Teilnehmer lehnten eine präventive Anpassung der antirheumatischen Therapie im Rahmen der SARS-CoV-2-Pandemie ab. Bei ambulanten Patienten unter Immunsuppression mit nachgewiesener COVID-19-Erkrankung würden mehr als 50 % der Teilnehmer folgende Therapien aussetzen: intravenöse hoch dosierte Steroide, Cyclophosphamid, Anti-CD20-Antikörper, sowie eine BAFF-, CTLA-4-, TNF-α-Blockade. Hingegen würden nichtsteroidale Antiphlogistika, Hydroxychloroquin (HCQ), orale Steroide, Mycophenolat, IL-1-Blockade sowie Immunglobuline (Ig) von >70 % der Kollegen weiter fortgeführt. Bei stationären Patienten mit COVID-19 würden insgesamt 74,6 % der Kollegen eine COVID-19-gerichtete Therapie erwägen. Bei stabilem Verlauf unter O_2_-Therapie (Stufe I) würden am häufigsten HCQ (18,3 %), Azithromycin (16,9 %) und Ig (9,9 %) in Betracht gezogen. Bei drohendem (Stufe II) bzw. manifestem Zytokinsturm (Stufe III) würden am häufigsten Anakinra (40,8 % bei Stufe II bzw. 46,5 % bei Stufe III), Tocilizumab (26,8 % bzw. 40,8 %), Steroide (25,4 % bzw. 33,8 %) und Remdesivir (29,6 % bzw. 38,0 %) eingesetzt. Von vielen Kollegen wurde betont, dass die Therapiestrategie individuell und der klinischen Situation entsprechend angepasst werden soll.

**Diskussion:**

Die Ergebnisse der Online-Umfrage sind vor dem Hintergrund einer aktuell in Deutschland niedrigen Prävalenz von COVID-19 zu sehen und spiegeln somit theoretische Überlegungen der Befragten wider. Da Kinder derzeit nicht im Fokus von prospektiven COVID-19-Studien stehen, scheint der kontinuierliche und kritische kollegiale Fachaustausch bei Therapieentscheidungen umso wichtiger zu sein.

## Abstract (en)

**Background:**

Reliable data on the course and treatment of pediatric COVID-19 (“corona virus disease 2019”) in immunosuppressed patients with rheumatic diseases are missing.

**Aim:**

Delineation of individual strategies of the members of the Society for Pediatric Rheumatology (GKJR) in cases of COVID-19.

**Methods:**

In May 2020 all GKJR members were invited to take part in an online survey. Opinion data regarding an approach using disease-modifying anti-rheumatic drugs (DMARD) in cases of COVID-19 as well as the readiness to use new therapeutic agents in patients in different stages of the disease were collected.

**Results:**

A total of 71 respondents (27.3% of all contacted pediatric rheumatologists) took part in the survey. Of these 28.2% had treated patients with COVID-19. Over 95% of the respondents did not support a preventive adaptation of the anti-rheumatic treatment during the SARS-CoV‑2 pandemic. In the case of outpatients under immunosuppression with proven COVID-19 more than 50% of the respondents would refrain from administering intravenous high-dose steroids, cyclophosphamide, anti-CD20 antibodies as well as BAFF, CTLA‑4 and TNF-alpha blockades. Conversely, >70% of the respondents would continue the treatment with nonsteroidal anti-inflammatory drugs, hydroxychloroquine (HCQ), oral steroids, mycophenolate, IL‑1 blockade and immunoglobulins (Ig). In the case of inpatients 74.6% of respondents would consider targeted COVID-19 treatment. In stable patients with oxygen treatment (stage I) HCQ (18.3%), azithromycin (16.9%) and Ig (9.9%) were most frequently used. In cases of early signs (stage II) or a manifest cytokine storm (stage III) anakinra (40.8% for stage II and 46.5% for stage III), tocilizumab (26.8% and 40.8%, respectively), steroids (25.4% and 33.8%, respectively) and remdesivir (29.6% and 38.0%, respectively) were most frequently used. The need for a personalized approach based on the current clinical situation was emphasized by many respondents.

**Conclusion:**

The currently low prevalence of COVID-19 in Germany limits the general clinical experience. Therefore, the presented results have to be interpreted with caution and mostly as hypothetical treatment considerations. It is to be expected that there will always be a limited amount of evidence on pediatric COVID-19; therefore, a continuous and critical exchange of expert opinions on the treatment strategies is important.

Die SARS-CoV-2-Pandemie stellt Kinderheumatologen und Pädiater allgemein vor große Herausforderungen, wenngleich in Deutschland bislang erfreulicherweise nur wenige schwere stationäre Fälle zu verzeichnen sind. Noch gibt es keine gesicherte Evidenz zum Umgang mit einer vorbestehenden immunsuppressiven Dauermedikation während der aktuellen SARS-CoV-2-Pandemie und keine konkreten Therapieempfehlungen für Kinder, die an COVID-19 erkranken. Eine der häufigsten Fragen in Gesprächen unter Kollegen ist aktuell: „Wie würdet Ihr das bei Euch in der Klinik eigentlich handhaben?“ Anhand der Ergebnisse dieser Online-Umfrage wird versucht, hierauf eine vorläufige Antwort zu geben.

## Hintergrund

Rheumatologen und Immunologen spielen eine zunehmend wichtige Rolle in der strategischen Planung des Vorgehens bei immunsupprimierten Patienten während der SARS-CoV-2-Pandemie und generell bei Patienten mit Hyperinflammationskomponente im Rahmen von COVID-19 („corona virus disease 2019“) [15, 20].

Das Robert Koch-Institut (RKI) dokumentierte in Deutschland bisher 7131 COVID-19-Fälle bei Kindern (<15 Jahre alt; Stand am 19.06.2020), entsprechend 3,75 % aller in Deutschland gemeldeten Erkrankungen [24]. Stationär behandlungsbedürftige Kinder mit COVID-19 werden in Deutschland über ein Register der Deutschen Gesellschaft für pädiatrische Infektiologie (DGPI) erfasst, an welches bislang 162 Patienten mit COVID-19-Erkrankung aus 84 Zentren (Stand in der 24. Kalenderwoche) gemeldet wurden. Dreizehn Prozent dieser Patienten waren intensivtherapiepflichtig [4, 10, 31]. Zudem werden SARS-CoV-2-Infektionen bei Kindern und Jugendlichen mit rheumatischen Grunderkrankungen anhand eines Zusatzmoduls der Kerndokumentation rheumakranker Kinder und Jugendlicher oder im BiKeR-Register erfasst. Darüber wurden bisher bundesweit insgesamt 16 SARS-CoV-2-Infektionen bei Patienten mit rheumatischen Grunderkrankungen registriert (Stand am 12.06.2020). Eines dieser Kinder musste stationär behandelt werden und ist bedauerlicherweise verstorben [5].

Nach derzeitigem Erkenntnisstand verlaufen Erkrankungen mit SARS-CoV‑2 bei gesunden Kindern häufiger asymptomatisch oder milder als bei älteren Personen [6, 14, 22, 31]. Obwohl Kinder mit rheumatischen Erkrankungen unter immunmodulatorischer und immunsuppressiver Therapie grundsätzlich als Risikopatienten bezüglich schwer verlaufender Virusinfektionen eingestuft werden [29, 30], handelt es sich bislang auch bei diesen Patienten in der überwiegenden Zahl der Fälle eher um milde COVID-19-Erkrankungen [17, 23]. Allerdings wurde kürzlich ein neues Hyperinflammationssyndrom („pediatric inflammatory multisystem syndrome“ [PIMS]; oder MIS‑C für „multisystem inflammatory syndrome in children“) mit variablem Erkrankungsverlauf beschrieben und in Verbindung mit SARS-CoV‑2 gebracht [12, 27].

Verschiedenste Stellungnahmen und Empfehlungen zum Umgang mit pädiatrischen COVID-19-Patienten wurden bereits veröffentlicht [2, 3, 7, 8, 11, 18, 26, 32]. Die rheumatologischen und immunologischen Fachgesellschaften haben Empfehlungen für die Behandlung von immunsupprimierten Patienten im Zusammenhang mit SARS-CoV‑2 publiziert [3, 9, 18, 26], welche hygienische und organisatorische Maßnahmen [3, 8, 26], den möglichen Umgang mit vorbestehenden immunmodulierenden und immunsuppressiven Therapien während der aktuellen Pandemie [9, 18] sowie mögliche medikamentöse Therapien bei milder bis schwer verlaufender COVID-19-Erkrankung betreffen [2, 7, 11]. Diese Stellungnahmen beruhen weitestgehend auf einer Extrapolation von Erfahrungen mit anderen, jedoch vergleichbaren infektiologischen und immunologisch/rheumatologischen Erkrankungen, kleinen Fallserien und somit letztendlich auf konsentierten Expertenmeinungen verschiedener Fachgesellschaften. Analog hierzu müssen im klinischen Alltag zahlreiche individuelle Therapieentscheidungen getroffen werden.

Der Online-Survey reflektiert das Meinungsbild der teilnehmenden deutschsprachigen Kinderrheumatologen zu folgenden Themen: a) Umgang mit vorbestehenden DMARD-Therapien bei ambulanten Patienten mit chronisch entzündlichen Erkrankungen, die an COVID-19 erkranken, und b) mögliche Therapiekonzepte bei stationären Patienten mit leichtem bis schwerstem Verlauf von COVID-19.

## Methodik

### Umfrage

Alle Mitglieder der Gesellschaft für Kinder- und Jugendrheumatologie (GKJR; Gesamtzahl 303 Personen, davon sind 260 ärztlich tätig und 171 als Kinderrheumatologen zertifiziert) wurden mittels E‑Mail-Verteiler zur Umfrage eingeladen. Die Umfrage wurde mittels SurveyMonkey® (www.surveymonkey.com) online gestellt und vom 11.05.2020 bis 19.05.2020 zur Beantwortung freigegeben. Erhoben wurden Daten über die Einrichtung der Teilnehmer, ihre Beschäftigungsdauer im Bereich der Kinderrheumatologie und ihre praktische Erfahrung bei der Betreuung von Patienten mit COVID-19. Abgefragt wurde der Umgang mit DMARDs im Falle ambulanter Patienten, die milde Symptome einer nachgewiesenen COVID-19-Erkrankung zeigten. Antwortmöglichkeiten umfassten, die Dauermedikation zu belassen, zu reduzieren oder auszusetzen. Die Option einer präemptiven DMARD-Reduktion im Rahmen der aktuellen COVID-19-Pandemie (ohne COVID-19-Erkrankung, SARS-CoV-2-Positivität oder Viruskontakt) wurde ebenfalls abgefragt. Ein zweiter Schwerpunkt bezog sich auf stationäre Patienten mit milder bis schwerster COVID-19-Erkrankung. Zunächst wurde ermittelt, ob eine andere COVID-19 gerichtete („off-label“) Therapie eingeleitet würde (nach ausführlicher und schriftlicher Aufklärung von Patienten/Eltern). Mögliche zur Behandlung von COVID-19 verwendete Medikamente wurden in einer Tabelle alphabetisch aufgelistet. Die Therapieauswahl sollte jeweils für einen „Standardpatienten“ bzw. einen „Patienten mit erhöhtem Risiko“ für einen schweren Verlauf einer Viruserkrankung (z. B. aufgrund einer relevanten Immundefizienz oder schweren kardiopulmonalen, nephrologischen bzw. neurodegenerativen Grunderkrankung) in unterschiedlichen COVID-19-Erkrankungsstadien (Stufe I: Pneumonie mit stationärem Aufenthalt und Sauerstoffbedarf; Stufe II: plus respiratorische Verschlechterung und/oder drohendem Zytokinsturm; Stufe III: kritisch kranker Patient mit „acute respiratory distress syndrome“ [ARDS] bzw. Multiorganversagen) getroffen werden. Als Kriterien für Stufe II der COVID-19-Erkrankung wurden folgende Parameter angegeben: *klinische Parameter*: ↑ Atemfrequenz, ↑ O_2_-Bedarf, ↑ pCO_2_; Röntgen Thorax/CT Thorax mit bilateralen Infiltraten, subpleuralen Milchglastrübungen oder Konsolidierung; *laborchemische Parameter*: ↓ Lymphozyten, ↓ Thrombozyten, ↑ IL‑6, ↑↑ CRP, ↑ PCT, ↑ IL-2R, ↑ Ferritin, ↑ LDH, ↑ Transaminasen, ↑ D‑Dimere, ↑ Troponin [11].

### Datenanalyse

Die statistische Analyse der Unterschiede zwischen den jeweiligen Subgruppen der Teilnehmer (unterteilt nach Art der Einrichtung, Beschäftigungsdauer und Erfahrung in der Behandlung von Patienten mit COVID-19) wurde mittels R (v 3.4.4) und dplyr (v 0.7.8) Software durchgeführt. „Double sided Fisher’s exact test“ und Cochran-Mantel-Haenszel-Test wurden angewendet.

## Ergebnisse

### Charakteristik der Umfrageteilnehmer (Tab. 1)

**Table 1:** 

*Typ der Einrichtung*	
Universitätsklinik	26 (36,6 %)
Nichtuniversitäre Klinik	27 (38,0 %)
Praxis	18 (25,4 %)
*Beschäftigungsdauer in der Kinderrheumatologie*	
<5 Jahre	5 (7,0 %)
5–10 Jahre	16 (22,5 %)
>10 Jahre	50 (70,4 %)
*Erfahrung in der Behandlung von COVID-19-Patienten*	
Ja	20 (28,2 %)
Nein	51 (71,8 %)

Die Umfrage wurde von 71 ärztlichen Kollegen der GKJR ausgefüllt, entsprechend 27,3 % aller ärztlichen GKJR-Mitglieder.

### Umgang mit DMARDs im Rahmen der COVID-19-Pandemie

Drei Teilnehmer der Umfrage (4,2 %), würden präventiv (d. h. ohne nachgewiesene SARS-CoV-2-Infektion) eine vorbestehende Dauertherapie bei Patienten mit klinisch inaktiver Grunderkrankung reduzieren bzw. absetzen. Diese 3 Kollegen würden v. a. eine aktuelle Steroidtherapie kritisch reevaluieren.

**Figure 1:**
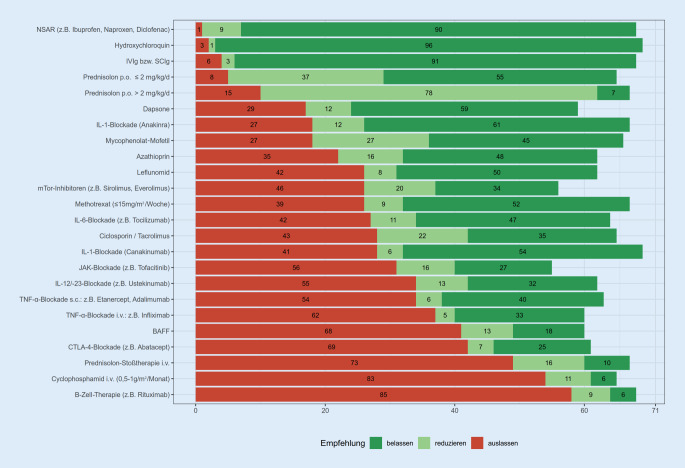

Die Abb. 1 zeigt den Umgang mit der antirheumatischen Dauermedikation bei Kindern und Jugendlichen, die mit Zeichen einer beginnenden COVID-19-Erkrankung (mit nachgewiesener SARS-CoV-2-Infektion) zunächst ambulant geführt werden könnten. Zirka 10 % der Teilnehmer haben betont, dass die Therapiestrategie in dieser Situation von der Schwere der SARS-CoV2-Infektion, Aktivität der Grunderkrankung, Dosierung und Kombination von DMARDs sowie der Vorgeschichte des Patienten abhängig wäre. Manche Teilnehmer haben sich zu Medikamenten, die sie selbst nie anwenden, nicht geäußert.

Befragte aus den nichtuniversitären Kliniken und niedergelassen Praxen gaben tendenziell etwas häufiger an, nachfolgende Medikamente bei ambulanter COVID-19-Erkrankung auszusetzen: subkutane TNF-α-Blockade (Nicht-Uniklinik: 15/27 und Praxis: 13/18 vs. Uniklinik: 6/26; *p* = 0,008), mTor-Inhibitoren (10/27 und 11/18 vs. 4/26; *p* = 0,014), IL-1-Blockade (Canakinumab: 13/27 und 10/18 vs. 5/26; *p* = 0,022; Anakinra: 7/27 und 8/18 vs. 3/26; *p* = 0,044); Mycophenolat-Mofetil (6/27 und 9/18 vs. 3/26; *p* = 0,022) und intravenöse und subkutane Immunglobuline (1/27 und 3/18 vs. 0/26; *p* = 0,025). Auch die Berufserfahrung hat Einfluss auf den Umgang mit einer vorbestehenden antirheumatischen Dauertherapie, so würden Kollegen mit längerer Berufserfahrung seltener eine subkutane TNF-α-Blockade bei einer ambulant zu führenden COVID-19-Erkrankung auslassen (Erfahrung <5 Jahre: 3/5, 5 bis 10 Jahre: 10/16 vs. >10 Jahre: 21/50; *p* = 0,006), jedoch würden sie häufiger eine intravenöse Therapie mit Cyclophosphamid (3/5 vs. 14/16 und 38/50; *p* = 0,030) oder eine Prednisolon-Stoßtherapie (3/5 und 11/16 vs. 35/50; *p* = 0,045) pausieren. Hingegen hatte die praktische Erfahrung in der Behandlung von COVID-19-Erkrankten keinen statistisch signifikanten Einfluss auf diese Therapieentscheidungen.

### Therapieansätze bei pädiatrischen Patienten mit COVID-19

**Figure 2:**
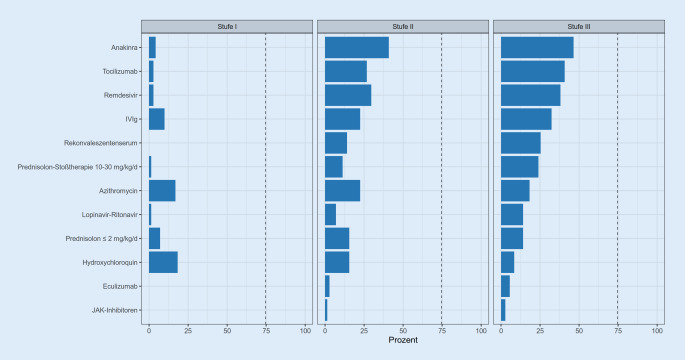

Eine COVID-19 gerichtete („off-label“) Therapie zusätzlich zu einer leitlinienkonformen Standardtherapie einer Pneumonie konnten sich 74,6 % der Teilnehmer der Umfrage vorstellen. Diese Entscheidung war unabhängig von Einrichtung, Berufserfahrung sowie praktischer Erfahrung in der Behandlung von COVID-19-Patienten. Das Gesamtergebnis zu möglichen Therapieansätzen ist in Abb. 2 dargestellt. Ein statistischer Unterschied zwischen Therapievorschlägen für Standard- und Risikopatienten wurde nicht festgestellt.

Zusätzlich zu den vorgegebenen Antworten wurde von einem Befragten der mögliche Einsatz von niedrig dosiertem Heparin und ggf. Defibrotid vorgeschlagen.

Sieben Teilnehmer der Umfrage würden eine COVID-19-gerichtete („off-label“) Therapie nur nach Rücksprache mit Kollegen aus einer universitären Einrichtung oder der Intensivstation evtl. einsetzen. Ein Teilnehmer würde intravenöse Immunglobuline nur dann anwenden, wenn das Präparat nachweislich spezifische Anti-SARS-CoV-2-Antikörper enthielte. Mehrere Kollegen hoben die Heterogenität des klinischen Verlaufs von COVID-19 hervor und betonten, dass eine Therapieentscheidung immer individuell der klinischen Situation angepasst erfolgen sollte.

## Diskussion

Der hier präsentierte Online-Survey gibt ein aktuelles Stimmungsbild der teilnehmenden deutschen Kinderrheumatologen zum Umgang mit Rheumapatienten während der SARS-CoV-2-Pandemie und der Behandlung von an COVID-19 erkrankten Kindern wieder. Im Einklang mit Empfehlungen nationaler und internationaler rheumatischer Fachgesellschaften [8, 18, 26] lehnen es >95 % der Teilnehmer der Umfrage ab, eine vorbestehende antirheumatische Dauertherapie präventiv (d. h. ohne nachgewiesene COVID-19-Infektion) zu reduzieren bzw. abzusetzen.

Im Fall von ambulanten Patienten mit beginnender COVID-19-Erkrankung haben sich fast alle Teilnehmer für eine unveränderte Fortsetzung der Therapie mit nichtsteroidalen Antirheumatika (NSAR), Hydroxychloroquin und subkutanen oder intravenösen Immunglobulinen geäußert. Dies steht im Einklang mit dem aktuellen Wissensstand. Bisher gibt es keine Hinweise für einen negativen Einfluss dieser Medikamente auf den COVID-19-Erkrankungsverlauf [1, 21]. Die Mehrheit der Kollegen würde auch eine vorbestehende orale Steroidtherapie weiterführen, allerdings eine höhere Dosierung von >2 mg/kg/Tag reduzieren. Die Fortsetzung einer Steroiddauertherapie wird im Hinblick auf eine potenzielle Nebennierenrindeninsuffizienz (bei einer Vorbehandlung >14 Tage) so auch von den rheumatischen Fachgesellschaften empfohlen [8, 9]. Hingegen würden 69 % der Teilnehmer eine hoch dosierte intravenöse Steroidstoßtherapie pausieren. Im Einklang mit den Ergebnissen zur Dauertherapie kann man spekulieren, dass diese Patienten dann alternativ eine niedrig dosierte Steroid-„bridging“-Therapie mit Prednisolon ≤2 mg/kg/Tag erhalten könnten. Die klare Empfehlung gegen eine Fortsetzung von B‑ und T‑Zell-wirksamen Medikamenten mit (immunologisch) langer Halbwertszeit wie Cyclophosphamid, JAK-Inhibitoren, Anti-CD20- und Anti-BAFF-Antikörpern sowie CTLA-4-IgG ist immunologisch nachvollziehbar [14]. Zudem würde der große Teil der Teilnehmer auch TNF-Inhibitoren und IL-12/23-Inhibitoren pausieren (s. Abb. 1). Hingegen spricht sich eine Mehrheit für eine Fortsetzung von Dapson, Mycophenolat, Leflunomid, Methotrexat, Azathioprin und Calcineurininhibitoren aus, wobei die jeweilige Medikation dann von einem Teil der Kollegen in reduzierter Dosis verabreicht würde. Es ist zu vermuten, dass die Subgruppe der teils stark antiproliferativen Medikamente wie Azathioprin, Mycophenolat und Calcineurininhibitoren aus Sorge vor einem schweren Schub z. B. bei Kollagenosen und Vaskulitiden weiter fortgesetzt würden. Bei COVID-erkrankten Patienten, die eine solche fortgesetzte antirheumatische Therapie erhalten, könnten engmaschige Laborkontrollen auf Blutbildveränderungen, Leber- und Nierentoxizität sowie im Einzelfall auch immunologische Parameter wie IgG, IgM und CD4-T-Zellen die Sicherheit der Anwendung erhöhen [25]. Die Mehrheit spricht sich auch gegen eine COVID-bedingte Beendigung einer Anti-IL-1- und Anti-IL-6-Therapie aus. Dies ist ein wichtiger Aspekt, da z. B. die Beendigung einer kurz wirksamen IL1-gerichteten Therapie wie Anakinra das Risiko eines Rebound-Phänomens in sich birgt und die zugrunde liegenden (systemischen) rheumatischen oder inflammatorischen Erkrankungen ein erhöhtes Risiko für ein Makrophagenaktivierungssyndrom (MAS) mit sich bringen, eine Komplikation die auch bei einigen COVID-19-Patienten beobachtet wurde [16, 19]. Bei Präparaten, die seltener bei Kindern mit rheumatischen Erkrankungen eingesetzt werden, wie z. B. Dapson, mTor-Inhibitoren oder JAK-Blockade, konnte die Unsicherheit der Umfrageteilnehmer dokumentiert werden, die sich durch eine relativ hohe Zahl an fehlenden Antworten ausdrückte.

Der Einsatz möglicher COVID-19-gerichteter („off-label“) Therapien in der Behandlung von Kindern und Jugendlichen ist umstritten. Dreiundfünfzig (74,6 %) der Kollegen könnten sich bereits im Frühstadium einer manifesten COVID-19-Erkrankung, bei stationärer Aufnahme wegen Sauerstoffbedarf, den Einsatz einer Off-label-Medikation vorstellen, wenn diese additiv zu einer leitlinienkonformen Pneumonietherapie und unter Berücksichtigung der individuellen klinischen Situation erfolgt. Mehr als zwei Drittel würden dieses Vorgehen erst im Stadium einer vorgeschrittenen Erkrankung befürworten, nach Beginn eines Zytokinsturms und/oder bei progredienter respiratorischer Insuffizienz (Stufe II und III der Erkrankung). Die Ausrichtung der COVID-19-gerichteten Therapie wandelt sich dabei mit zunehmender Schwere der Erkrankung. Eine Therapie mit Hydroxychloroquin und Azithromycin wurde dabei von den Teilnehmern der Studie eher in der frühen Phase der Erkrankung als relevante Option eingestuft. Die Wertigkeit der Steroidtherapie nimmt mit der Schwere der Erkrankung zu, im Stadium III würden insgesamt 33,8 % (24/71) der Kollegen den Einsatz von oralen oder intravenösen Steroiden befürworten. Intravenöse Immunglobuline (IVIG) haben einen Stellenwert in allen 3 Krankheitsstadien. Die Daten für Steroide und Immunglobuline decken sich mit aktuellen Real-life-Behandlungsdaten weltweit [13]. Mit zunehmender Schwere der Erkrankung und drohendem oder bereits laufendem Zytokinsturm neigen die Kollegen zum Einsatz einer IL-1- bzw. IL-6-Blockade sowie zur Gabe von Konvaleszentenplasma. Aus den vorgeschlagenen antiviralen Präparaten wurde nur Remdesivir von den Befragten in Betracht gezogen. Remdesivir würde dabei erst später im Verlauf eingesetzt, ob es dabei kombiniert mit einer Zytokinblockade erfolgen würde, bleibt aufgrund der Fragestellung des Surveys zwar offen, wird aktuell aber in einer globalen randomisierten doppelblinden placebokontrollierten Studie untersucht [28]. Eine Kombination aus antiviraler Medikation und/oder Immunglobulingabe oder Konvaleszentenplasma auf der einen Seite mit einer antiinflammatorischen Therapie aus Zytokininhibitoren und/oder Steroiden auf der anderen Seite könnte eine mögliche Therapiestrategie darstellen und wurde in Einzelfällen auch bereits publiziert [16]. Aktuell haben Eculizumab und JAK-Inhibitoren für die Teilnehmer der Studie keine Bedeutung in der Behandlung von COVID-19. Wir konnten keinen statistisch signifikanten Unterschied zwischen den in Betracht gezogenen Therapien für Standard- und Risikopatienten feststellen. Aktuelle Studien zum frühen vs. späten Einsatz COVID-19-gerichteter Therapien im Krankheitsverlauf werden derzeit für Hydroxychloroquin, Remdesivir und Tocilizumab durchgeführt (https://clinicaltrials.gov) und könnten in Zukunft ggf. eine Basis für differenzierte Empfehlung für Hochrisikopatienten schaffen.

### Limitationen

In der Umfrage konnten nur eingeschränkt therapeutische Entscheidungsprozesse abgebildet werden. Die Heterogenität der klinischen Situation und mögliche (Kombinations‑)Therapien konnte nicht berücksichtig werden. Dank der sehr niedrigen COVID-19-Prävalenz in Deutschland ist die praktische Erfahrung der Befragten niedrig, und die Aussagen basieren großenteils auf hypothetischen Überlegungen. Die Schlussfolgerungen müssen mit Vorsicht interpretiert werden und sollen nicht als eine Empfehlung gelten.

## Ausblick

Aktuell geht die Ausbreitungsgeschwindigkeit der SARS-CoV-2-Infektion in Deutschland und Europa zurück [24], jedoch ist die Wahrscheinlichkeit, dass mit steigender Prävalenz auch die Anzahl schwerer Fälle steigt, weiterhin hoch, zudem ist ein rekurrierender Verlauf nicht ausgeschlossen. Wir sollten für eine längere Auseinandersetzung mit der Erkrankung vorbereitet sein. Die steigende klinische Erfahrung und die Daten aus kontrollierten Studien werden es ermöglichen, den therapeutischen Entscheidungsprozess zu präzisieren. Deshalb ist eine Wiederholung der Umfrage im späteren Verlauf der Pandemie geplant.

## Fazit für die Praxis

- Die Evidenzlage bezüglich des Umgangs mit immunsuppressiven Basistherapien bei Patienten mit rheumatologischen Erkrankungen im Rahmen der COVID-19-Pandemie ist aktuell nicht ausreichend.
- Klinische Studien werden im Verlauf eine Präzisierung der therapeutischen Ansätze, auch nach individuellen Risikofaktoren, ermöglichen; es ist aber zu erwarten, dass die Datenlage betreffend die Therapie von COVID-19-Erkrankung bei Kindern und Jugendlichen eingeschränkt bleiben wird.
- Die medikamentöse Therapie muss im Rahmen einer COVID-19-Erkrankung individuell angepasst werden, eine Pauschalisierung der Therapieansätze ist nicht möglich; ein kontinuierlicher interdisziplinär kollegialer Fachaustausch ist nötig.
